# Occupational Health Complaints Among Physical Education Teachers: A Scoping Review of Musculoskeletal, Vocal, Hearing, Respiratory, Injury-Related, Cardiometabolic, and Work-Limiting Outcomes

**DOI:** 10.3390/jcm15207838

**Published:** 2026-10-11

**Authors:** Jadwiga Herba, James Chmiel, Mirela Niedzielska, Marta Stępień-Słodkowska

**Affiliations:** 1 Faculty of Physical Culture and Health, Institute of Physical Culture Sciences, University of Szczecin, Al. Piastów 40B blok 6, 71-065 Szczecin, Poland; jadwiga.herba@phd.usz.edu.pl (J.H.);; 2 Department of the History of Medicine and Medical Ethics, Pomeranian Medical University, ul. Rybacka 1, 70-204 Szczecin, Poland

**Keywords:** physical education teachers, occupational health, health complaints, musculoskeletal disorders, work-related injuries, voice disorders, dysphonia, noise exposure, hearing complaints, respiratory symptoms, cardiometabolic risk, work ability

## Abstract

**Background/Objectives:** Physical education teachers play an important role in school health promotion, but their own occupational health has received limited attention. Their work involves movement demonstration, prolonged standing, equipment handling, sport participation, vocal projection, noise exposure, and outdoor or indoor environmental demands. This scoping review aimed to map evidence on occupational health complaints and disorders among physical education teachers and closely related exercise-teaching professionals meeting a functional occupational definition. **Methods:** A scoping review was conducted using PubMed/MEDLINE, Scopus, Web of Science, SPORTDiscus, and ERIC, with ScienceDirect as an additional publisher platform. Google Scholar, ResearchGate, and reference-list screening were used as supplementary discovery sources. Records from database inception to 30 May 2026 were considered without language restrictions. School-based physical education teachers were the primary population; related gym/fitness, aquatic, university, and student-teaching professionals were eligible only when their occupational role centered on structured physical education, sport, exercise, or movement instruction. Empirical quantitative, qualitative, mixed-methods, and eligible grey literature reports were included when they contained extractable original data. Data were charted by study characteristics, outcome domain, measurement method, and main findings; because of marked heterogeneity, a narrative synthesis was performed. **Results:** Thirty-eight studies met the inclusion criteria. School-based physical education teachers predominated, while a smaller number of studies represented related instructional settings. Musculoskeletal disorders and injury-related complaints were the most frequently reported outcomes, especially affecting the lower back, knees, ankles/feet, shoulders, neck, and hips. Vocal and laryngeal problems, hearing and noise-related complaints, respiratory and upper-airway symptoms, and cardiometabolic risk factors were also identified. Across domains, observational studies repeatedly reported associations involving physical loading, high vocal demand, noise, environmental conditions, and age or professional seniority. Because most evidence was cross-sectional or descriptive, these patterns do not establish causal relationships. **Conclusions:** The mapped literature identifies a multidimensional set of occupational health concerns in physical education teaching and closely related instructional work. Generalization is strongest for school-based physical education teachers, while non-school groups provide context-specific supporting evidence. Prospective, longitudinal, and intervention evidence remains limited. Future research should use longitudinal designs, standardized outcome definitions, objective clinical and environmental assessments, and clearer exposure measurement. Preventive strategies such as ergonomic training, injury prevention, vocal-health education, acoustic improvements, noise monitoring, respiratory-risk reduction, and cardiometabolic screening warrant further evaluation in this occupational population.

## 1. Introduction

Schools are increasingly understood not only as educational institutions, but also as health-promoting environments in which learning, physical activity, wellbeing, and occupational conditions interact. The World Health Organization and UNESCO define a health-promoting school as a setting that strengthens its capacity as a healthy place for living, learning, and working [1]. Within this framework, physical education has a particularly important role because it supports movement competence, physical literacy, health-related knowledge, social development, and lifelong engagement in physical activity [2]. These priorities are also aligned with global public health recommendations emphasizing the importance of regular physical activity for children, adolescents, adults, and older adults [3]. Physical education teachers therefore occupy a central position in school health promotion: they are expected to model active lifestyles, deliver movement-based education, prevent sedentary behaviour, and support students’ physical and psychosocial development.

However, the occupational health of physical education teachers themselves has received considerably less attention than their role in promoting students’ health. This imbalance is important because physical education teaching differs substantially from classroom-based teaching. It commonly involves prolonged standing, movement demonstration, equipment handling, supervision in large or noisy spaces, outdoor work, exposure to variable temperature and humidity, participation in sport activities, vocal projection, and management of injury risk. As a result, physical education teachers may be exposed to a combination of biomechanical, vocal, auditory, respiratory, environmental, and cardiometabolic stressors. These exposures suggest that their health profile may not be adequately captured by the general teacher-health literature or by studies of sedentary occupations.

Musculoskeletal disorders are one of the most plausible occupational health concerns in this group. General teacher populations already show a high prevalence of musculoskeletal symptoms, particularly in the back, neck, shoulders, and upper limbs [4,5]. In the broader occupational-health literature, work-related musculoskeletal disorders have been linked to heavy physical work, repetitive movements, awkward postures, forceful exertions, lifting, high psychosocial demands, and other workplace exposures [6,7]. Prolonged standing has also been associated with low back and lower-limb symptoms [8], while occupational activities such as lifting, kneeling, squatting, climbing, and standing have been associated with higher odds of knee osteoarthritis [9]. These mechanisms are highly relevant to physical education teaching, where demonstration, assisting pupils, moving equipment, rapid movement transitions, and sport-related loading may accumulate across years of professional practice.

Vocal health represents another major concern. Teachers are among the most frequently studied professional voice users, and the wider literature shows that voice disorders are more common in teachers than in the general population [10,11,12]. Occupational voice problems are linked to prolonged speaking, high vocal intensity, insufficient vocal rest, background noise, poor room acoustics, dry air, respiratory irritation, stress, and limited access to voice-care training [11,12,13]. Physical education teachers may be especially vulnerable because they often teach in gymnasiums, sports halls, swimming pools, outdoor fields, and other acoustically difficult environments where they must speak loudly over background noise and across long distances. Thus, vocal symptoms such as hoarseness, dysphonia, aphonia, dry throat, vocal fatigue, and laryngeal irritation may represent predictable consequences of the occupational communication demands of physical education.

Hearing and noise-related complaints are also relevant to this profession. Occupational noise exposure is a recognized cause of hearing loss, tinnitus, and reduced auditory function [14]. Beyond hearing loss itself, noise has non-auditory health effects, including annoyance, sleep disturbance, stress responses, impaired concentration, and possible cardiovascular consequences [15]. Physical education environments can be acoustically challenging because sports halls, gyms, pools, and fitness rooms often combine reverberation, large groups, shouting, whistles, impact sounds, music, and poor sound absorption. Noise may therefore affect physical education teachers directly through auditory strain and indirectly by increasing vocal load. This interaction between noise and voice demand is especially important because it may create a cycle in which noisy environments force teachers to raise their voices, while repeated vocal effort increases the risk of vocal fatigue and dysphonia.

Respiratory and upper-airway complaints may arise from several occupational pathways. Schools are complex indoor environments, and poor indoor air quality has been associated with respiratory symptoms, asthma, rhinitis, discomfort, absenteeism, and reduced productivity in students and staff [16,17]. Physical education teachers may additionally work in environments with specific respiratory exposures, including cold outdoor air, dust from sports halls, humidity, cleaning agents, pool disinfectant by-products, and repeated close contact with students during periods of viral transmission. In aquatic environments, airborne chloramines and related disinfection by-products have been linked to occupational asthma and respiratory symptoms in swimming-pool workers [18,19]. These mechanisms suggest that respiratory complaints in physical education teachers should not be viewed only as common infections, but also as potentially related to environmental and workplace exposures.

Cardiometabolic health is another important but potentially overlooked issue. Physical education teachers are often assumed to be protected from obesity, hypertension, dyslipidemia, and cardiovascular risk because their work is associated with sport and movement. However, broader occupational-health research indicates that occupational physical activity does not always produce the same health benefits as structured leisure-time physical activity [20]. The “physical activity paradox” proposes that leisure-time physical activity is generally cardioprotective, whereas occupational physical activity may be prolonged, repetitive, insufficiently restorative, and performed under stress or time pressure [20]. Therefore, physical education teachers may still develop cardiometabolic risk factors, especially when occupational movement is not accompanied by adequate recovery, balanced training, healthy diet, sleep, and preventive screening.

Despite the plausibility of these occupational risks, the literature on health complaints among physical education teachers remains fragmented. Existing studies differ in their populations, countries, settings, age groups, teaching levels, outcome definitions, measurement methods, and comparison groups. Some studies focus on musculoskeletal pain, while others focus on injuries, voice, noise, respiratory complaints, cardiovascular risk, BMI, or general work ability. This heterogeneity makes it difficult to draw a single pooled estimate of disease prevalence, but it makes the topic particularly appropriate for a scoping review. Scoping reviews are recommended when the aim is to map the extent, range, and nature of evidence; clarify concepts; identify knowledge gaps; and determine whether more focused systematic reviews or meta-analyses are warranted.

A scoping review of occupational health complaints in this field is justified for three main reasons. First, school-based physical education teachers constitute the primary population of interest because their work combines teaching, movement demonstration, sport participation, environmental exposure, and high vocal demand. At the same time, occupational titles and work settings vary internationally, and some physical educators, gym/fitness instructors, aquatic instructors, and other exercise-teaching professionals perform closely related instructional tasks. The present review therefore used a functional occupational definition that allowed these related groups to be included when structured physical education, sport, exercise, or movement instruction formed a central part of their work, while preserving the setting of each study during synthesis. Second, the available evidence is dispersed across multiple outcome domains, including musculoskeletal, injury-related, vocal, hearing, respiratory, cardiometabolic, and other work-limiting conditions. A broad mapping approach is needed to integrate these domains without assuming that prevalence estimates are interchangeable across school, fitness, aquatic, or other instructional settings. Third, the methodological diversity of the field—ranging from self-report questionnaires to clinical examination, audiometry, environmental measurements, administrative records, and qualitative interviews—makes scoping synthesis more suitable than immediate meta-analysis. By identifying what has been studied, how it has been measured, and where evidence gaps remain, this review can provide a foundation for future longitudinal research, occupational screening, preventive interventions, and workplace-health policies.

Accordingly, the aim of this scoping review was to map and synthesize the available evidence on occupational health complaints and disorders among physical education teachers, with school-based teachers as the primary population and closely related exercise-teaching professionals included when they met the functional eligibility definition, with particular attention to musculoskeletal, vocal, hearing, respiratory, injury-related, cardiometabolic, and other work-limiting outcomes.

## 2. Materials and Methods

### 2.1. Review Design

This study was designed as a scoping review aimed at mapping the available evidence on occupational health complaints and disorders among physical education teachers. A scoping review approach was selected because the literature on this topic is heterogeneous in terms of study design, population, occupational setting, outcome definition, and measurement method. The purpose of the review was therefore not to estimate a pooled prevalence or treatment effect, but to identify the range of health outcomes studied in physical education teachers, describe how these outcomes were assessed, and summarize the main patterns reported across studies.

### 2.2. Research Question

The review was guided by the following research question: what occupational health complaints and disorders have been reported among physical education teachers and closely related exercise-teaching professionals who meet the functional population definition specified below?

More specifically, the review aimed to identify and describe evidence concerning musculoskeletal disorders, vocal and laryngeal disorders, hearing and noise-related complaints, respiratory and upper-airway complaints, injury-related outcomes, cardiometabolic risk factors, and other work-limiting health problems in physical education teachers.

### 2.3. Eligibility Criteria

Eligibility criteria were defined using the Population–Concept–Context framework. The primary population of interest comprised school-based physical education teachers. Because occupational titles and professional structures differ across countries and settings, eligibility was based on occupational function rather than job title alone. Physical educators, physical education instructors, assistant physical education teachers, and physical education student teachers were eligible when they were engaged in teaching practice. Fitness instructors, gym-based physical education professionals, aquatic physical education teachers, and similar exercise-teaching professionals were eligible only when their core occupational role involved structured physical education, sport, exercise, or movement instruction and the relevant health data were attributable to that instructional work. Mixed teacher samples were included only when results for physical education teachers or another eligible instructional subgroup were reported separately or when that subgroup was clearly identifiable.

The concept of interest was occupational health complaints, disorders, diseases, injuries, or physical health risk factors. Eligible outcomes included musculoskeletal pain and disorders, acute or chronic injuries, osteoarthrosis, vocal and laryngeal symptoms or findings, hearing problems and noise-related symptoms, respiratory and upper-airway complaints, infectious complaints, cardiometabolic risk factors, dermatological outcomes, work-limiting disorders, and other physical health problems. Studies focused solely on pedagogy, student outcomes, professional identity, physical-activity habits, or psychological constructs without a physical health outcome were excluded.

The context included schools, colleges, universities, gymnasiums, health clubs, aquatic facilities, sport settings, and fitness settings in which physical education or exercise instruction was performed occupationally. Related non-school settings were included to map context-specific exposure and health patterns, not because they were assumed to be equivalent to school physical education. School, university, gym/health-club, aquatic, fitness, and student-teaching contexts were retained as distinct study characteristics during synthesis. No restrictions were imposed on country, educational level, participant age, sex, years of professional experience, workload, or other demographic or occupational characteristics. These variables were treated as descriptive or explanatory study characteristics rather than as selection criteria.

Original empirical quantitative, qualitative, and mixed-methods reports were eligible when sufficient methodological information and extractable outcome data were available. Eligible publication formats included peer-reviewed journal articles and, where they contained original empirical data, theses/dissertations and scholarly book chapters. Reviews, editorials, commentaries, conference abstracts without sufficient data, and reports without extractable results for physical education teachers were excluded.

### 2.4. Information Sources

The search covered records from database inception to 30 May 2026. Primary bibliographic database searches were conducted in PubMed/MEDLINE, Scopus, Web of Science, SPORTDiscus, and ERIC. ScienceDirect was searched as an additional publisher platform. Google Scholar was used as a supplementary academic search engine, and reference lists of relevant publications were screened manually for additional eligible reports.

ResearchGate was not treated as a bibliographic database and did not replace the established database searches. It was used only as a supplementary discovery and document-access source to locate potentially relevant publications or studies in the grey literature identified through keyword, author, title, or citation searching. Any potentially eligible record identified through ResearchGate, Google Scholar, or reference-list screening was added to the same master record set, deduplicated against database records, and subjected to the same title/abstract and full-text eligibility assessment.

Dedicated Spanish-language or regional databases such as Dialnet, ENFISPO, and SciELO were not searched systematically. The systematic search was deliberately centered on broad biomedical, multidisciplinary, sport, and education databases that allowed a consistent strategy across countries and disciplines; omission of regional databases was therefore a pragmatic scope decision rather than a language-based exclusion. To reduce the risk of missing regional or non-English evidence, publications in any language remained eligible and supplementary discovery was undertaken through Google Scholar, ResearchGate, and reference-list screening. Nevertheless, the literature indexed only in regional databases may have been missed, and this issue is addressed explicitly in the limitations.

### 2.5. Search Strategy

The search strategy combined terms describing the occupational group with terms describing occupational health outcomes. Search strings were adapted to the syntax and functionality of each source. The core Boolean structure was as follows: (“physical education teacher*” OR “PE teacher*” OR “physical educator*” OR “physical education instructor*” OR “sports teacher*” OR “gym teacher*” OR “fitness instructor*” OR “aquatic instructor*”) AND (“occupational health” OR “health complaint*” OR “work-related disorder*” OR “musculoskeletal disorder*” OR “low back pain” OR “neck pain” OR “shoulder pain” OR “knee pain” OR injur* OR osteoarthritis OR “voice disorder*” OR dysphonia OR aphonia OR hoarseness OR “vocal fatigue” OR “hearing loss” OR tinnitus OR “noise exposure” OR respiratory OR asthma OR bronchitis OR rhinitis OR cardiovascular OR hypertension OR obesity OR BMI OR “work ability”).

Truncation, phrase searching, and Boolean operators were used where supported. No publication-year restriction was applied. No language restriction was imposed at the search or eligibility stage: publications in English, Spanish, Portuguese, French, Polish, and other languages were eligible when sufficient methodological and outcome information was accessible for reliable extraction. Non-English records were screened against the same inclusion and exclusion criteria as English-language records, and publication language was not itself a reason for exclusion.

### 2.6. Study Selection

All records identified through database, platform, and supplementary searches were consolidated into a single master set. Duplicate records were removed by matching DOI when available and by cross-checking title, author, and publication year. Records identified through ResearchGate, Google Scholar, or reference-list screening were therefore not treated as a separate evidence stream after deduplication.

Study selection was conducted in two stages. Titles and abstracts were first screened for relevance to the review question. Full texts of potentially eligible reports were then assessed against the Population–Concept–Context criteria and the publication-type criteria described above. Studies were included when they examined physical education teachers or a clearly separable related professional group and reported at least one occupational health complaint, disorder, injury, disease category, or physical health risk factor. Studies were excluded when the PE-teacher subgroup could not be separated, no relevant health outcome was reported, or insufficient information was available for extraction.

Uncertain records were resolved by re-reading the full text and reapplying the eligibility criteria. The selection process is reported in a PRISMA-ScR flow (Supplementary Table S2) diagram showing the numbers identified, deduplicated, screened, excluded, assessed at full text, and included.

### 2.7. Data Charting

Data were extracted using a structured data-charting form developed for this review. The extracted information included author, year of publication, country, study design, setting, sample size, participant characteristics, sex distribution, age, teaching level, professional experience, comparison group when present, health outcome domain, measurement method, main findings, and occupational factors associated with the reported outcomes.

Health outcomes were grouped into the following categories: musculoskeletal disorders and injury-related complaints; vocal and laryngeal disorders; hearing and noise-related complaints; respiratory, upper-airway, and infectious complaints; cardiometabolic outcomes and risk factors; and other occupational health complaints or work-limiting disorders. When a study reported outcomes across more than one domain, data were extracted separately for each relevant category.

Because this was a scoping review, data charting was iterative. Categories were refined during extraction to reflect the range of outcomes identified in the literature. For example, some studies reported broad otorhinolaryngological or throat-related categories that overlapped with vocal, respiratory, and hearing domains. In such cases, outcomes were assigned to the most relevant category based on the description provided by the original study.

### 2.8. Data Synthesis

A descriptive and narrative synthesis was conducted. First, study characteristics were summarized according to country, occupational setting, sample size, sex distribution, age, teaching level, and study design. Second, findings were organized by disorder group. For each outcome domain, the synthesis described the types of complaints reported, anatomical or clinical distribution where applicable, measurement approaches, comparison groups, and occupational factors associated with the outcomes.

Due to heterogeneity in study designs, populations, outcome definitions, recall periods, and measurement tools, no meta-analysis was performed. The results are therefore presented as a structured mapping of the evidence rather than as pooled estimates. Study design was explicitly considered when interpreting findings: cross-sectional, descriptive, retrospective, record-based, and qualitative studies were used primarily to map prevalence, co-occurrence, and reported associations; prospective or longitudinal studies were used to inform temporal patterns; and intervention effects were discussed separately when supported by randomized evidence. Accordingly, occupational exposures identified in observational studies are described as correlates or plausible contributors rather than established causes. Numerical results were reported when available, but direct quantitative comparison between studies was avoided unless studies used similar outcome definitions or comparable measurement approaches.

## 3. Results

**Figure 1:**
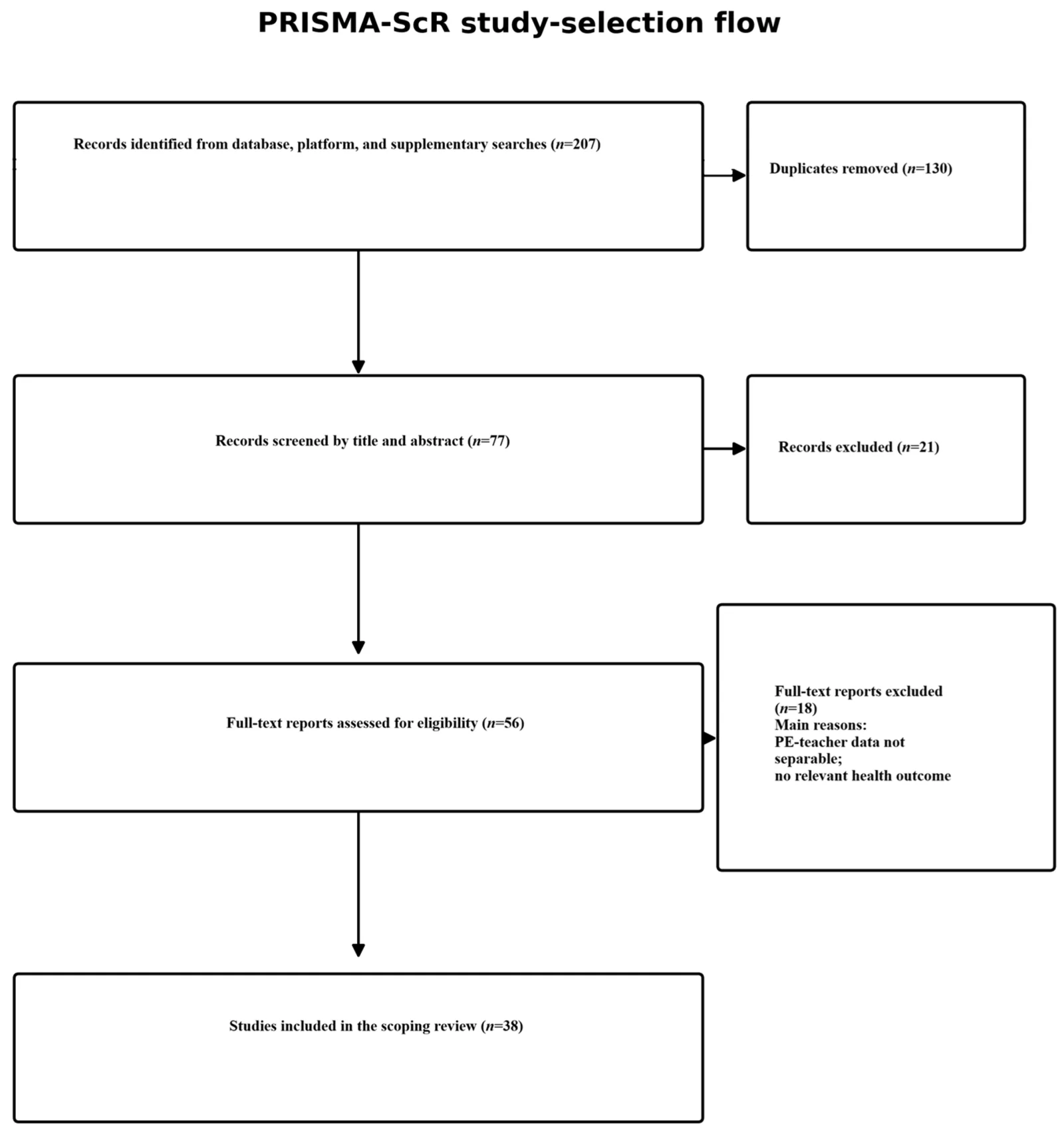
PRISMA-ScR flow diagram of study selection.

Figure 1 summarizes study selection. In total, 207 records were identified across database, platform, and supplementary searches. After removal of 130 duplicates, 77 records underwent title/abstract screening and 21 were excluded. Fifty-six full-text reports were assessed; 18 were excluded for one of two eligibility reasons: PE-teacher or other eligible instructional-professional data could not be separated from broader samples, or the report did not contain an eligible occupational physical-health outcome. These exclusion categories and their operational definitions are detailed in Supplementary Table S1. Thirty-eight studies met the eligibility criteria and were included in the review [21,22,23,24,25,26,27,28,29,30,31,32,33,34,35,36,37,38,39,40,41,42,43,44,45,46,47,48,49,50,51,52,53,54,55,56,57,58].

### 3.1. Participant Characteristics

The 38 included studies examined heterogeneous groups of physical education teachers and related physical education professionals in school, university, gym, aquatic, and fitness settings. Evidence came from Europe, Latin America, North America, Africa, and Asia, with particularly frequent contributions from Slovenia, Brazil, Finland, Turkey, Poland, Canada/Quebec, and Spanish- or Portuguese-speaking settings [21,22,23,24,25,26,27,28,29,30,31,32,33,34,35,36,37,38,39,40,41,42,43,44,45,46,47,48,49,50,51,52,53,54,55,56,57,58]. Most studies were cross-sectional or descriptive observational studies. Temporal evidence was limited to prospective injury surveillance [22] and longitudinal repeated voice assessment [32], while only one randomized controlled trial directly evaluated an injury-prevention intervention [41]. The remaining evidence included retrospective questionnaire data [24], qualitative interviews [37], and studies using institutional records or clinical/environmental measurements [30,38,39,40,47,52,54]. This distribution of designs is important for interpretation because most findings describe prevalence, co-occurrence, or association rather than temporal or causal effects.

Sample sizes ranged from 6–12 participants in several qualitative or exposure studies [37,38,55,56] to several hundred teachers in larger occupational-health and work-ability studies [21,25,27,28,35,36]. School-based physical education teachers predominated, but some studies examined gym or fitness instructors [23,39,42,43], aquatic teachers [44], student teachers [32], or broader physical/health education professionals [45]. These related groups met the functional eligibility criterion because their professional roles centered on structured movement or exercise instruction and shared relevant occupational tasks such as demonstration, physical participation, voice use, or work in sport facilities. However, the settings were not treated as interchangeable: fitness and indoor-cycling studies may involve amplified-music exposure, aquatic teaching introduces pool-specific environmental conditions, and student teachers have substantially shorter occupational exposure [32,39,43,44]. Consequently, these studies broaden the map of possible occupational health concerns but should not be interpreted as direct prevalence estimates for school-based physical education teachers. Measurement methods ranged from symptom questionnaires and injury histories to audiometry, sound-level monitoring, laryngoscopy, dermatological examination, blood-pressure and anthropometric assessment, and heart-rate-variability recording [30,31,38,39,42,47,52,54].

Age, sex distribution, teaching level, professional experience, and workload varied substantially. Some samples were predominantly male [23,25,29,30,34,47,53], whereas others were female-predominant or female-only [32,35]. Professional exposure ranged from student teaching or a few years of employment to more than 30 years in older teachers [23,24,32,37]. Several studies included non-PE teachers, students, or population referents as comparison groups [21,22,24,29,40,42,48]. These demographic and occupational characteristics were used to contextualize findings; they were not eligibility criteria. Detailed study-level characteristics are provided in Table 1.

### 3.2. Musculoskeletal Disorders and Injury-Related Complaints

Musculoskeletal disorders and injury-related complaints were the most consistently reported outcomes. Across countries and settings, the lower back, cervical region, knees, ankles/feet, shoulders, hips, and upper limbs were repeatedly affected [21,22,23,24,25,26,27,28,29,33,34,35,36,37,40,41,43,44,45,46,56,57,58]. Comparative evidence generally indicated a higher burden in PE teachers than in non-PE teachers or population referents. For example, Belgian PE teachers sustained 1.23 injuries per teacher-year versus 0.78 in non-PE teachers [22], and a Turkish record-based study reported musculoskeletal diseases in 24.5% of PE teachers versus 11.5% of other teachers [40].

Low-back complaints were especially prominent. Prevalence reached 63% among 562 male Greek PE teachers, with associations involving assistance of pupils in flexed postures, equipment lifting, high weekly teaching hours, and lack of personal training [25]. In Slovenia, low-back pain affected approximately half of PE teachers and increased with age [28], while surveys from Hyderabad, Burkina Faso, Brazil, Colombia, and Argentina also identified lumbar symptoms as a recurrent problem [26,34,44,56,58]. Neck, upper-back, shoulder, elbow, and wrist/hand complaints were less consistently dominant but were repeatedly reported and sometimes caused activity limitation [23,26,28,37].

Knee and ankle/foot disorders formed a second major pattern. Swedish PE teachers had more knee injury, symptomatic knee osteoarthrosis, surgery, and knee-related sick leave than population referents even after adjustment for relevant covariates [24]. In Slovenia, serious career injuries were concentrated in the ankle/foot and knee, and 61.1% of injured teachers reported adapting their teaching afterward [27]. Quebec studies likewise identified lower-limb injuries, sprains/strains, tendinitis, back/neck lesions, medical follow-up, physical limitation, and work absence [29,45]. Work-ability studies reinforced the functional importance of these problems: among Finnish teachers reporting a work-limiting condition, musculoskeletal disorders accounted for 71%, particularly knee and back problems [35].

Across the predominantly cross-sectional and descriptive studies, reported correlates included age or professional seniority, prolonged standing, repeated demonstration, assisting pupils, lifting or transporting equipment, awkward postures, high weekly teaching hours, working across multiple sites, participation in sport outside teaching, and environmental hazards such as slippery or inadequate surfaces [25,27,28,29,33,34,35,36,37,44,46,56,57,58]. These associations identify candidate occupational factors but do not establish temporal sequence or causality. The prospective injury-surveillance study [22] provides stronger evidence for differences in injury incidence between PE and non-PE teachers, but it does not isolate a single causal occupational exposure. Aquatic and gym-based settings added context-specific loads, while older teachers described modifying demonstrations and participation in response to pain or reduced physical capacity [37,44].

Only one included randomized trial directly tested prevention. A multifactorial programme incorporating warm-up, cooling down, stretching, core stability, balance, functional strength, correct movement performance, and footwear was associated with a lower sports- or work-related injury rate in the intervention group than in controls (0.49 vs. 1.14 injuries per 1000 exposure hours), with the clearest effect on non-contact lower-limb injuries [41]. Given the modest final sample, this trial provides preliminary intervention evidence that at least part of the injury burden may be modifiable, but replication is needed.

### 3.3. Vocal and Laryngeal Disorders

Vocal and laryngeal problems were another recurrent occupational-health domain, consistent with the need to speak loudly and continuously in gymnasiums, sports halls, pools, outdoor spaces, and music-based fitness environments. Reported outcomes included hoarseness, dysphonia, aphonia, dry or sore throat, vocal fatigue, effortful speech, voice failure, and laryngeal abnormalities [21,28,31,32,36,40,43,54,55,58].

Large questionnaire studies indicated a substantial symptom burden. In Slovenia, dysphonia was reported by 37.7% of men and 52.4% of women and was more frequent in primary- than secondary-school PE teachers; aphonia was less common but also more frequent in women [28]. In a Brazilian sample, approximately two thirds of men and more than 70% of women reported vocal complaints, most commonly dry throat and hoarseness; intensive voice use was the most frequently perceived cause [31]. A small school-based study similarly found that 7 of 10 teachers exceeded a Voice Symptom Scale risk cutoff and that high-intensity, near-constant voice use was common [55].

Objective and longitudinal findings provided complementary evidence to the self-report literature. Seven female PE student teachers showed changes in acoustic and aerodynamic voice measures over a 14-week teaching semester, accompanied by mid-semester hoarseness, strain, raspiness, and episodes of voice loss [32]. In 30 active PE teachers examined by flexible nasopharyngolaryngoscopy, laryngeal hyperemia was observed in 96.66%, hypokinesia in 40%, and bilateral vocal-fold nodules in 26.66% [54]. These findings show that reported voice problems can coincide with measurable functional or structural findings; however, the longitudinal evidence came from only seven student teachers, while the laryngoscopic study was cross-sectional, so neither provides a basis for broad causal generalization.

Context-specific studies also linked vocal complaints with noisy indoor cycling environments, outdoor exposure, and conditions severe enough to limit work ability [36,43,58]. Taken together, the evidence suggests an interaction between vocal load, acoustically unfavorable environments, and environmental irritation; however, study designs were mostly cross-sectional and outcome definitions varied considerably.

### 3.4. Hearing and Noise-Related Complaints

Hearing and noise-related complaints were studied less often than musculoskeletal or vocal outcomes, but findings were consistent across school gymnasiums, fitness academies, health clubs, and indoor cycling settings. Outcomes included hearing difficulty, tinnitus, muffled hearing, sound intolerance, perceived post-work hearing changes, audiometric abnormalities, headache, irritability, insomnia, and concentration difficulties [21,28,29,38,39,42,43]. In Slovenia, auditory problems increased markedly with age, from 10.9% in teachers younger than 35 years to 43.0% in those older than 45 years; major auditory impairment showed a similar age gradient [28].

Environmental measurements confirmed that PE professionals may work in acoustically demanding spaces. In Brazilian gymnasiums, mean levels were below an 85-dB (A) 8-h occupational limit but often exceeded comfort recommendations, with peaks of up to 105 dB (A); tinnitus, perceived hearing change, headache, and class disruption were common [38]. Portuguese school and health-club measurements reached approximately 84–85 dB (A) in schools and around 90–92 dB (A) in several fitness classes, and exposed professionals reported more hearing difficulty, muffled hearing, sound intolerance, headache, and irritability than controls [42].

The clearest audiometric signal came from 32 Brazilian fitness teachers exposed to amplified music. Mean sound pressure was 101.4 dB (A), 50% had an audiometric notch in at least one ear, and 9.37% had audiometric abnormalities; sound intolerance and hearing difficulty were each reported by 43.8% [39]. Indoor cycling studies likewise documented sound levels around 100 dB (A) together with hearing- and throat-related complaints [43].

Overall, the auditory evidence is suggestive rather than definitive because samples were often small, cumulative occupational exposure was not measured uniformly, and symptom or audiometric outcomes were generally assessed cross-sectionally. The convergence of objective noise measurements, auditory symptoms, and audiometric changes nevertheless identifies high-noise PE and fitness environments as settings in which hearing conservation and acoustic control warrant attention, without establishing occupational noise as the cause of each observed auditory outcome.

### 3.5. Respiratory, Upper-Airway, and Infectious Complaints

Respiratory, upper-airway, and infectious complaints were reported less systematically and were usually embedded within broader occupational-health questionnaires rather than assessed through clinical respiratory testing. Across studies, reported outcomes included colds, influenza-like illness, sore or dry throat, rhinitis, sinusitis, otitis, bronchitis, asthma, shortness of breath, allergy, and other ENT-related complaints [21,28,35,36,40,43,44,58]. This heterogeneity limits direct prevalence comparisons across studies.

In Slovenia, common cold was frequent in teachers overall and was also reported among PE teachers without a clear association with sex, age, or teaching level [21,28]. Respiratory disorders represented 11% of work-limiting conditions among Finnish PE teachers [35], whereas 2.9% of Turkish teachers reported respiratory or throat/voice conditions severe enough to limit work, including bronchitis, asthma, shortness of breath, and recurrent throat problems [36]. Fitness and aquatic environments showed a broader symptom cluster: indoor bike instructors frequently reported throat problems and flu [43], and among aquatic teachers, 58.70% reported at least one disease or health problem, including flu, throat problems, sinusitis, allergy, rhinitis, and otitis [44].

Outdoor and institutional-record studies also identified recurrent ENT and respiratory complaints [40,58]. The Argentine outdoor-teaching study described exposure to cold, wind, dust, rain, and inadequate infrastructure alongside recurrent colds/rhinitis, sore throat, flu, bronchospasm, otitis, allergy, sinusitis, and asthma [58]. Because most evidence was self-reported and causal exposure assessment was limited, these findings should be interpreted as signals of possible occupational burden rather than as proof of a specific respiratory mechanism.

### 3.6. Cardiometabolic Outcomes and Risk Factors

Cardiometabolic outcomes were less frequently studied, but the available evidence shows that professional involvement in sport and physical activity does not eliminate cardiovascular or metabolic risk. Studies assessed BMI, overweight/obesity, blood pressure, smoking, lipid or dietary fat indicators, physical activity, and knowledge of chronic disease risk factors [30,40,47,48,49,50,51]. Across several datasets, men tended to have a less favorable body mass or cardiovascular risk profile than women [30,48,49].

Among 191 Croatian PE teachers, both sexes were more physically active than the general employed population, yet 21.4% smoked and male teachers had more excess body weight, higher systolic blood pressure, and less favorable cholesterol/fat intake indicators. Mild systolic hypertension was present in 32.3% of men versus 9.2% of women, and the combined cardiovascular risk score was higher in men [30]. In Benin, approximately 35% of male assistant PE teachers had high blood pressure, with anthropometric and atherogenic markers contributing to risk [47]. By contrast, a Turkish record-based study found similar frequencies of cardiovascular disease in PE and other teachers [40].

Body-mass findings were consistent with substantial subgroup heterogeneity. In Poland, working PE teachers had higher BMI than PE students, and overweight was especially frequent among male teachers [48]; a second Polish study likewise found overweight/obesity concentrated in men [49]. In Indonesia, 52 of 124 teachers were classified as overweight or obese [50]. In Brazil, 39.1% of teachers were classified as overweight/obese and 43.6% were insufficiently active during leisure time, despite generally good knowledge of major behavioral risk factors [51].

These studies do not establish that PE teaching causes cardiometabolic disease, but they challenge the assumption that a physically active occupation is uniformly protective. Age, sex, adiposity, smoking, recovery, diet, and the distinction between occupational and leisure-time activity remain important contextual factors when interpreting cardiometabolic health in this profession.

### 3.7. Other Occupational Health Complaints and Work-Limiting Disorders

Several studies reported additional occupational-health outcomes that did not fit the main domains, including headache, dermatological conditions, urinary complaints, gastrointestinal or hematological diagnoses, broad neurological/”brain and nerve” categories, sleep or irritability symptoms, psychological complaints, and reduced work ability [21,28,35,36,38,39,40,52,53,57,58]. These outcomes were generally secondary findings and were less consistently defined, so their occupational specificity is uncertain.

Dermatological evidence most directly concerned a plausible workplace exposure. In Costa Rica, clinical examination of 47 PE educators with substantial outdoor sun exposure identified dermatoheliosis, pigmentary changes, nevi, actinic keratoses, and lesions considered suggestive of basal-cell carcinoma [52]. In Mexico, 28.2% of 131 teachers reported previous sunburn, most often affecting the face and arms, while regular dermatological monitoring was uncommon [53]. These observations justify attention to solar exposure and photoprotection in outdoor PE work, but the cross-sectional evidence does not establish the magnitude of any occupation-specific causal effect.

Work ability provided an integrative functional outcome. In Finland, 31% of PE teachers reported at least one disorder that interfered with work, with musculoskeletal problems accounting for 71% of those conditions; older teachers reported lower work ability and described workload, physical fitness, mental stress, working conditions, and voice as future concerns [35]. In Turkey, 8.1% reported a work-limiting injury or disease, again predominantly musculoskeletal, followed by respiratory or throat/voice problems [36]. Qualitative interviews with older Finnish teachers showed that health limitations could change demonstration practices, participation in lessons, confidence, and career planning even without complete work absence [37].

Other signals should be interpreted cautiously. Noise-exposed teachers reported headache, insomnia, irritability, and dizziness [38,39]; institutional records from Turkey used broad disease categories that are difficult to map to specific occupational mechanisms [40]; and the two included Argentine theses reported broad self-reported physical and general health burdens but had limitations in reporting consistency [57,58]. These findings support a multidimensional view of PE-teacher health while also showing where better-defined outcomes and stronger study designs are needed.

## 4. Discussion

This scoping review maps a broad and multidimensional spectrum of occupational health complaints in physical education teaching. The most frequently reported domains were musculoskeletal disorders and injury-related complaints, followed by vocal and laryngeal disorders, hearing and noise-related symptoms, respiratory and upper-airway complaints, cardiometabolic risk factors, and several other work-limiting health problems. School-based physical education teachers formed the core evidence base. Smaller groups of gym/fitness instructors, aquatic teachers, student teachers, and related professionals were included because their work met the functional criterion of structured movement or exercise instruction, but these populations are not interchangeable with school teachers. Their inclusion broadens the range of occupational exposures mapped by the review, while conclusions about school physical education should rely most heavily on school-based studies. Interpretation must also reflect study design: most evidence was cross-sectional or descriptive and can identify prevalence and associations but cannot establish temporality or causation; prospective or longitudinal evidence was limited to studies [22,32], and only study [41] provided randomized intervention evidence. Accordingly, the mechanistic explanations discussed below should be read as plausible interpretations of observed patterns unless stronger designs are specifically noted.

The predominance of musculoskeletal complaints is mechanistically plausible, but most evidence linking specific occupational tasks to symptoms was observational. Physical education teachers repeatedly perform movements that may include demonstration, lifting or moving equipment, assisting pupils, rapid changes in direction, prolonged standing, and participation in physically demanding activities. These exposures correspond to recognized ergonomic factors associated with work-related musculoskeletal disorders, including heavy physical work, repetitive movement, awkward posture, forceful exertion, and high physical workload [6,7,59,60]. Within the reviewed studies, low-back pain was reported in association with several such tasks and workload characteristics, but the predominantly cross-sectional designs cannot determine whether the occupational exposure preceded the disorder. The frequent occurrence of low-back pain is therefore compatible with a contribution from combined trunk flexion, manual handling, sudden movements, and prolonged standing rather than evidence of a single causal factor. Broader occupational research has associated prolonged standing with low-back and lower-extremity symptoms, especially when combined with other physical exposures [8,61], which provides mechanistic context but does not by itself prove causation in PE teachers.

The concentration of complaints in the lower back, knees, ankles/feet, hips, and shoulders also suggests that the musculoskeletal profile reported in physical education teachers may differ from that of less physically demanding teaching occupations. In general teacher populations, musculoskeletal symptoms are often concentrated in the neck, shoulders, and lower back, reflecting classroom posture, computer work, prolonged standing, and psychosocial workload [4,5]. In contrast, the present review identified a more sports- and movement-related distribution, with substantial reporting of knee, ankle/foot, hip, and injury-related outcomes. This pattern is consistent with the broader occupational and sports-medicine literature in which knee loading, kneeling, squatting, lifting, climbing, and previous knee injuries are associated with knee osteoarthritis and long-term knee symptoms [9,62,63]. However, the reviewed cross-sectional and retrospective studies cannot establish progression from PE-teaching exposure to degenerative joint disease. Knee injuries, knee osteoarthrosis, and knee-related work limitations should therefore be regarded as a potential concern for long-term joint health rather than evidence that PE teaching itself causes degenerative disease.

The injury-related findings are especially important because injuries were repeatedly reported alongside occupational consequences such as sick leave, medical follow-up, reduced physical status, altered teaching methods, and reduced ability to demonstrate movements. This is consistent with the general concept that musculoskeletal disorders can reduce work capacity, productivity, and long-term employability. For physical education teachers, such consequences may be particularly important because demonstration, mobility, agility, and confidence in movement are part of the core professional role. A knee injury, shoulder disorder, or lumbar pain episode may therefore coincide with limitations in the ability to model movement, supervise safety, assist pupils, and participate in lessons. The prospective injury-surveillance evidence [22] strengthens the temporal description of injury occurrence, whereas most evidence on longer-term work consequences remains observational or retrospective.

Vocal and laryngeal disorders represented the second major occupational health domain. This is consistent with the broader literature identifying teachers as professional voice users with a higher prevalence of voice disorders than many other occupational groups [10,11,64]. Teaching requires prolonged voice use, often with limited vocal rest, and physical education teaching adds candidate exposure factors such as large spaces, gymnasiums, outdoor fields, background noise, distance from pupils, and the frequent need to raise the voice. These conditions plausibly increase vocal intensity and phonatory effort and are therefore relevant to vocal fatigue, hoarseness, dysphonia, aphonia, and phonotraumatic lesions such as vocal fold nodules [11,12,65]. However, apart from the small longitudinal study of seven student teachers [32], the included evidence was mainly cross-sectional. The findings are therefore compatible with an exposure-response hypothesis, but they do not establish that repeated high-intensity voice use causes a temporal progression from functional symptoms to structural laryngeal abnormalities.

The sex differences observed in several vocal outcomes are also consistent with the previous teacher-voice literature. Systematic reviews and epidemiological studies have repeatedly shown that women report voice problems more frequently than men, although the reasons are probably multifactorial [10,11,65]. Possible explanations include anatomical and physiological differences in vocal fold size and vibratory frequency, differences in teaching context, hormonal factors, and different thresholds for symptom reporting or healthcare seeking. However, sex should not be interpreted as a purely biological risk factor. In occupational voice disorders, biological susceptibility interacts with modifiable workplace factors such as noise, room acoustics, class size, vocal rest, hydration, training in voice use, and access to amplification [11,12]. This is important because it suggests that prevention should focus not only on individual vocal hygiene but also on environmental and organizational modifications.

Hearing and noise-related complaints formed a closely related occupational domain. The reviewed studies showed that physical education professionals may work in environments where sound pressure levels are high enough to interfere with communication and, in some settings, approach or exceed occupational noise thresholds. The broader occupational-noise literature supports the relevance of these findings. Occupational noise exposure is a well-established cause of noise-induced hearing loss and tinnitus, and systematic review evidence suggests that a meaningful proportion of adult hearing loss is attributable to workplace noise [14]. NIOSH recommends an occupational noise exposure limit of 85 dB (A) averaged over an 8-h workday, and repeated exposure at or above this level is considered sufficient to require hearing-loss prevention measures [66]. This is particularly relevant for fitness, indoor cycling, gymnasium, and amplified-music settings, where sound levels may be intermittent but intense. Within the included review evidence, however, objective noise measurements mainly characterized exposure environments, whereas the links between those exposures and individual symptoms or audiometric findings were generally cross-sectional.

The presence of tinnitus, muffled hearing, hearing difficulty, intolerance to loud sounds, and audiometric notches in some included studies suggests that auditory symptoms among physical education professionals should not be dismissed as minor discomfort. Noise-induced hearing loss typically affects high frequencies first, and audiometric notches around 3–6 kHz are commonly used as markers of possible noise-related cochlear injury [67,68]. At the same time, the reviewed evidence also described extra-auditory symptoms such as headache, irritability, concentration difficulties, sleep changes, and fatigue. This is consistent with broader evidence that noise has auditory and non-auditory effects, including annoyance, stress responses, sleep disturbance, cognitive interference, and cardiovascular strain [69]. In physical education settings, noise could therefore plausibly contribute to a dual burden by coinciding with auditory symptoms and greater vocal effort, but the reviewed cross-sectional studies do not establish the direction or magnitude of these causal pathways.

Respiratory, upper-airway, and infectious complaints were less consistently assessed, but they remain occupationally relevant. The reviewed studies reported colds, flu, rhinitis, sinusitis, bronchitis, asthma, shortness of breath, sore throat, angina, and other upper-airway complaints. These symptoms may be compatible with several overlapping pathways. Teachers work in close contact with children and adolescents; physical education may also be delivered in cold, humid, dusty, poorly ventilated, or outdoor environments; and aquatic settings may involve exposure to chlorination by-products such as chloramines. Studies in indoor swimming-pool workers and lifeguards have linked chloramine exposure with asthma-like symptoms and occupational asthma [13,19,70]. Accordingly, air quality, ventilation, humidity, temperature changes, disinfectant by-products, and infection exposure are plausible contextual mechanisms, but the predominantly self-reported and cross-sectional PE-teacher evidence does not demonstrate that any specific exposure caused the reported respiratory outcomes.

School indoor air quality may also be relevant. Broader studies of school environments have linked poor classroom air quality, dampness, pollutants, and inadequate ventilation with asthma, rhinitis, cough, and other respiratory symptoms [16,71]. Although many of these studies focus on pupils rather than teachers, the same environments are also workplaces for adults who may spend years or decades in these buildings. For physical education teachers, exposure may be more variable than for classroom teachers because teaching can occur in gyms, sports halls, outdoor fields, swimming pools, changing rooms, and storage areas. This variability may explain why respiratory outcomes were heterogeneous across the included studies and why they were often reported as broad categories rather than precise clinical diagnoses.

Cardiometabolic outcomes were less frequently studied but are important because they challenge the assumption that physical education teachers are automatically protected from cardiovascular risk. The reviewed studies showed that elevated BMI, overweight, obesity, hypertension, smoking, adverse lipid indicators, and increased cardiometabolic-risk scores were present in some samples, especially among men. This is consistent with the broader cardiometabolic literature showing that obesity, hypertension, dyslipidemia, smoking, and physical inactivity remain major cardiovascular risk factors [72,73]. The findings are also compatible with the “physical activity paradox,” which proposes that occupational physical activity may not produce the same cardiovascular benefits as structured leisure-time physical activity [20,74,75]. Occupational physical activity is often prolonged, repetitive, insufficiently recuperative, and performed under time pressure or environmental stress, whereas leisure-time physical activity is more often voluntary, structured, and followed by recovery.

This distinction is important for interpreting cardiometabolic risk in physical education teachers. Their work may involve movement, standing, demonstration, and physical participation, but this does not necessarily equal adequate aerobic training, balanced resistance exercise, recovery, sleep, nutrition, or cardioprotective leisure-time activity. Moreover, age, male sex, adiposity, smoking, stress, and cumulative workload may offset the benefits of occupational movement. The reviewed finding that neck circumference was associated with high blood pressure is also consistent with broader evidence that neck circumference can serve as a marker of upper-body adiposity and cardiometabolic risk [76]. Therefore, cardiometabolic screening in physical education teachers should not be neglected simply because they are professionally associated with sport and exercise.

The additional complaints identified in the results, including headache, urinary tract infections, dermatological diseases, neurological or brain-and-nerve disease categories, gastrointestinal complaints, insomnia, irritability, dizziness, and general work-limiting conditions, should be interpreted cautiously. These outcomes were often reported as broad administrative or self-reported categories, and their occupational specificity is less clear than for musculoskeletal, vocal, hearing, or respiratory disorders. Some complaints may plausibly coexist with workplace exposures—for example, headache with noise, dehydration, or environmental conditions; gastrointestinal complaints with stress or irregular routines; and dermatological complaints with sweating, sun exposure, disinfectants, or sports-facility environments—but the included evidence does not establish these explanations. These observations are therefore best treated as hypothesis-generating signals that support a multisystem view of PE-teacher health and identify priorities for more focused study.

Overall, the findings are compatible with a cumulative-exposure framework for occupational health in physical education teaching, but this framework should be treated as a hypothesis-generating synthesis rather than a causal model established by the included studies. Within such a framework, biomechanical load, injury risk, vocal strain, noise, environmental respiratory irritants, infectious exposure, and cardiometabolic factors may coexist and potentially interact. For example, noise may coincide with increased vocal demand; injury may reduce opportunities for physical activity; chronic pain may accompany reduced work ability; respiratory symptoms may limit teaching endurance; and age or professional seniority may be associated with a greater cumulative burden of some complaints. These proposed interactions could help organize future longitudinal hypotheses, but they should not be interpreted as demonstrated causal chains.

From a practical perspective, the review identifies potential prevention priorities, but direct evidence that specific interventions are effective in physical education teachers is limited. The only included randomized trial [41] found a lower injury rate after a multifactorial injury-prevention programme, providing preliminary intervention evidence in the musculoskeletal domain. Other proposed measures—such as safe demonstration strategies, equipment-handling support, conditioning and recovery planning, voice training or amplification, acoustic modification, noise monitoring, ventilation improvement, pool-air-quality control, and cardiometabolic screening—should be regarded as risk-management implications based on the mapped exposure patterns and broader occupational-health principles rather than as interventions proven effective in this population. Their effectiveness should be evaluated in prospective and controlled studies.

Several methodological limitations should be emphasized. First, the population breadth was deliberate but introduces heterogeneity. School-based physical education teachers predominated, whereas some studies included gym/fitness instructors, aquatic teachers, student teachers, or broader physical/health education professionals. These groups share structured movement or exercise instruction but differ in exposure intensity, acoustic conditions, environmental agents, teaching schedules, and duration of professional exposure. Their inclusion increases the breadth of the scoping map but reduces direct comparability; findings from non-school groups should therefore be interpreted as context-specific and should not be generalized automatically to school-based PE teachers. Second, most included studies were cross-sectional and relied on self-reported symptoms, limiting causal inference and introducing possible recall bias. Only a small part of the evidence base provided prospective or longitudinal follow-up [22,32], and only one randomized trial directly evaluated an intervention [41]. Outcome definitions, recall periods, and measurement tools varied substantially, and several voice, noise, aquatic, and clinical studies used small samples. Comparators were inconsistent, and adjustment for age, sex, workload, previous injury, leisure sport, and non-occupational exposure was variable. The review also included original empirical studies in the grey literature, including two theses [57,58], because the scoping objective was to map the available evidence; these sources provide relevant data but generally offer less standardized reporting than peer-reviewed journal articles. Finally, although no language restriction was applied, dedicated regional databases such as Dialnet, ENFISPO, and SciELO were not systematically searched. Spanish, Portuguese, French, and other non-English reports could still be identified through the broader databases, supplementary search platforms, and citation searching; however, the literature indexed exclusively in regional databases may have been missed. This may have underrepresented locally published evidence, particularly from Latin American and Iberian sources, and may have influenced the geographic distribution of the mapped literature. Future reviews should prospectively include relevant regional databases and document source-specific record counts.

Future research should examine trajectories across the teaching career and explicitly separate exposure from age and professional seniority. Cross-sectional age or seniority gradients in the reviewed literature suggest that knee disorders, low-back pain, hearing problems, and work-ability limitations may be more common in older or more experienced teachers, but only longitudinal follow-up can determine whether occupational exposure precedes and predicts later disability. Longitudinal studies could test whether early injuries or voice symptoms predict subsequent teaching adaptation, sickness absence, or premature exit from the profession while controlling for leisure sport, previous injury, sex, age, and other confounders. It would also be valuable to test preventive interventions, such as voice amplification, acoustic treatment, injury-prevention programmes, ergonomic redesign, physical conditioning, and routine cardiometabolic screening. Such studies would move the field from descriptive occupational epidemiology toward stronger evidence for prevention and occupational-health policy.

## 5. Conclusions

This scoping review identified recurring occupational-health concerns across musculoskeletal and injury-related, vocal/laryngeal, hearing/noise, respiratory/upper-airway, and cardiometabolic domains, with musculoskeletal and injury-related complaints reported most consistently. Because the literature was predominantly cross-sectional or retrospective, these findings describe burden and associations rather than demonstrating that occupational exposures cause long-term disease; prospective and intervention evidence remains limited.

Accordingly, conclusions are most applicable to school-based physical education teachers, who formed the core evidence base. Findings from gym/fitness, aquatic, student-teaching, and other related instructional groups should be interpreted as context-specific rather than generalized directly to school-based PE teachers. The evidence supports treating physical education teaching as a distinct occupational-health context while avoiding causal attribution of individual long-term outcomes to the occupation itself.

The evidence base remains heterogeneous. Most studies used cross-sectional designs and self-reported outcomes, while only limited prospective or longitudinal evidence and one randomized intervention trial were available. Fewer studies applied clinical examination, audiometry, laryngoscopy, physiological measurement, environmental assessment, or prospective injury registration. Future research should therefore prioritize longitudinal designs, standardized outcome definitions, objective health assessments, clearer measurement of occupational exposures, and analytic control of important non-occupational factors. More attention should be paid to sex differences, teaching level, years of professional experience, school infrastructure, acoustic conditions, outdoor and aquatic environments, workload, recovery, and preventive occupational-health practices.

From a practical perspective, the findings justify consideration of targeted prevention and workplace-health strategies for physical education teachers, while recognizing that most proposed strategies have not yet been tested directly in this population. Potential measures include ergonomic training, injury-prevention programmes, access to physiotherapy or occupational-health screening, vocal-health education, acoustic improvements in sports facilities, use of voice amplification where appropriate, monitoring of occupational noise, respiratory-risk reduction in specific environments, and cardiometabolic screening despite the physically active nature of the profession. Further controlled and longitudinal studies are needed to determine which strategies improve health, work ability, and career sustainability.

## Figures and Tables

**Table 1:** Characteristics of included studies examining occupational health conditions among physical education teachers and related exercise-teaching professionals.

Study/Setting	Design and Sample	Domain(s)	Assessment/Data Source	Main Review—Relevant Findings
[21] Slovenia, school teachers	Cross-sectional; 858 teachers, including 340 PETs	MSK, VOC, AUD, RESP/general complaints	Chronic Health Disorders Questionnaire; logistic regression	PETs showed the highest occupational health-risk profile, especially for lower back, neck, hip, knee, foot, elbow and hearing problems.
[22] Belgium, secondary schools	Prospective cohort; 103 PE vs. 58 non-PE teachers; 43 weeks	INJ, MSK	Baseline questionnaire; weekly online injury registration	PE teachers had more injuries than non-PE teachers: 1.23 vs. 0.78 injuries/teacher/year; lower limbs, knee and back/neck were prominent.
[23] Brazil, gyms	Cross-sectional; 39 gym-based PE teachers	MSK	Online questionnaire; 27-region symptom diagram	Symptoms were more frequent in women; arm and knee pain reached 50% among women; pain was associated with previous-year injury.
[24] Sweden	Retrospective questionnaire study; PE-teacher graduates vs. population referents	MSK, WORK	Postal questionnaire; prevalence ratios	PE teachers had higher knee injury, symptomatic knee osteoarthrosis, knee surgery and knee-related sick leave; female PE teachers also had higher hip osteoarthrosis.
[25] Greece, Athens	Cross-sectional; 562 male PE teachers	MSK	Questionnaire on occupational demands and low back pain	Low back pain prevalence was 63%; risk factors included helping students in flexed postures, lifting gym equipment, >35 teaching h/week and no personal training.
[26] Hyderabad	Cross-sectional; 100 PE teachers/educators	MSK	Nordic Musculoskeletal Questionnaire	Twelve-month symptoms were most common in the lower back, shoulder, wrist/hand and neck; shoulder symptoms most often limited activity.
[27] Slovenia, primary/secondary schools	Cross-sectional; 468 PE teachers	INJ, MSK, WORK	Self-administered questionnaire on serious injuries	Serious career injuries were common: 65.1% of men and 55.0% of women; ankle/foot and knee injuries predominated; 61.1% adapted teaching after injury.
[28] Slovenia, primary/secondary schools	Cross-sectional; 468 PE teachers	MSK, VOC, AUD, RESP/general	Self-administered chronic health-problem questionnaire	Lower back pain affected about half of teachers; dysphonia, cervical spine problems, hearing problems and age-related joint disorders were common.
[29] Canada, Quebec	Cross-sectional; 314 PE teachers plus 19 classroom teachers	INJ, MSK, AUD, WORK	Self-administered questionnaire	Recent and chronic lesions were frequent; back/neck, ankle-foot and knee were common sites; injuries led to medical follow-up, pain, limitations and work absence.
[30] Croatia	Cross-sectional; 191 PE teachers	CARD	Baecke physical activity questionnaire; Wilson–Jungner CVD risk questionnaire	Male PE teachers had higher cardiovascular risk scores, more excess body weight, higher systolic blood pressure and less favorable cholesterol/fat-intake profile than women.
[31] Brazil	Cross-sectional; 46 PE teachers	VOC; autonomic physiology	Vocal Handicap Index; HRV recording	Vocal complaints were frequent; dry throat and hoarseness were common; organic VHI scores correlated negatively with parasympathetic HRV indices.
[32] USA, student teaching	Longitudinal repeated-measures; 7 female PE student teachers	VOC	Acoustic/aerodynamic voice measures; VHI; self-reports	Teaching semester was associated with changes in fundamental frequency, jitter, shimmer, subglottic pressure and subjective vocal fatigue/hoarseness.
[33] Spain, Jaén	Cross-sectional; 50 secondary-school PE teachers	INJ, MSK, WORK	Original self-administered questionnaire	Thirty percent reported work-related physical pathology; ankle sprain and low back pain were the most frequent; some injuries caused prolonged sick leave.
[34] Burkina Faso, Ouagadougou	Cross-sectional; 165 PE teachers	MSK	Adapted occupational MSD questionnaire	Overall MSD prevalence was 77.58%; hip, lumbar region and knees/legs were most affected; seniority and health history were associated with MSDs.
[35] Finland	Cross-sectional; 655 PE teachers	WORK, MSK, RESP	Work Ability Questionnaire; open disease categories	Thirty-one percent reported a disease/disorder limiting work ability; 71% of these were MSK, mainly knee and back problems.
[36] Turkey	Mixed-method/cross-sectional; 407 PE teachers	WORK, MSK, RESP/VOC	Turkish Work Ability Questionnaire; open responses	Work ability was generally high, but 8.1% reported limiting injury/disease; MSK problems were most common, followed by respiratory/throat/voice-related problems.
[37] Finland	Qualitative interviews; 6 PE teachers aged >55 years	MSK, WORK	In-depth interviews; thematic analysis	All teachers described MSK/physical-functioning problems affecting demonstrations, participation, confidence and long-term work ability.
[38] Brazil, gymnasiums	Cross-sectional exposure study; 12 PE teachers	AUD, headache, concentration/sleep symptoms	Objective noise measurement; discomfort questionnaire	Mean gym noise often exceeded comfort recommendations; strong discomfort was common for class disruption and headache; tinnitus and perceived hearing change were frequent.
[39] Brazil, fitness academy	Cross-sectional; 32 fitness/PE teachers	AUD, extra-auditory symptoms	Noise measurement; questionnaire; pure-tone audiometry	Mean SPL was 101.4 dB(A); 50% had audiometric notch; intolerance to intense sounds, hearing difficulty, headache and insomnia were common.
[40] Turkey	Descriptive screening; 110 PE vs. 113 other teachers	MSK, ENT/VOC, CARD, GI, DERM	Institutional disease records classified by physicians	MSK, brain/nerve, dermatological, hematological and GI diseases were more frequent in PE teachers; ENT diseases were very common but borderline vs. controls.
[41] Belgium, secondary schools	Randomized controlled trial; 55 PE teachers	INJ prevention, MSK	Weekly injury registration; prevention intervention	Multifactorial prevention reduced injury rate: 0.49 vs. 1.14 injuries/1000 h exposure; strongest effect for non-contact lower-limb injuries.
[42] Portugal, schools/health clubs	Cross-sectional exposure study; 48 PE professionals plus 52 controls	AUD, extra-auditory symptoms	Sound-level measurements; questionnaire	PE/fitness professionals had higher noise exposure and more hearing difficulties, muffled hearing, sound intolerance, headaches and irritability than controls.
[43] Brazil, indoor cycling gyms	Cross-sectional; 15 PE teachers	AUD, VOC/RESP, MSK, fatigue	Noise measurement; questionnaire; SRQ-20; Borg scale	Indoor-bike noise reached hazardous levels; throat problems, flu, hearing/ear problems and back aches were frequent.
[44] Brazil, aquatic activities	Cross-sectional; 184 PE teachers	MSK, RESP/VOC, skin/eye, INJ, psychological distress	Questionnaire; SRQ-20; Borg scale	Pain affected 52.72%, especially lumbar and knee pain; throat problems, sinusitis, skin/eye problems, otitis and work accidents were also reported.
[45] Canada, Quebec	Descriptive cross-sectional; 181 PE/health education teachers	INJ, MSK, WORK	Online questionnaire on physical activity and injuries	Most teachers reported at least one injury; many injuries occurred during personal physical activity or work and affected teaching capacity.
[46] France, secondary schools	Cross-sectional ergonomic survey; 218 PE/sport teachers	WORK, MSK, fatigue, sleep	Questionnaire; subgroup and logistic analyses	Despite generally good perceived physical condition, many reported health problems, joint trauma/pain, surgery risk, sickness absence, fatigue and difficulty continuing work.
[47] Benin	Cross-sectional; 123 male assistant PE teachers	CARD	Blood pressure, anthropometry, lipid and inflammatory markers	High blood pressure affected about 35%; increased neck circumference, waist circumference and atherogenic index were common.
[48] Poland	Cross-sectional; 127 PE teachers and 173 PE students	CARD/body mass, health behaviours	Health Behavior Inventory; IPAQ; self-reported BMI	PE teachers had higher BMI than students; overweight was common, especially among male teachers.
[49] Poland	Cross-sectional; 127 PE teachers	CARD/body mass, health behaviours	Health Behavior Inventory; body/self measures; BMI	BMI showed strong sex differences; overweight/obesity was common in men, while underweight appeared mainly among women.
[50] Indonesia	Descriptive case study; 124 PE teachers	CARD/body mass/nutritional status	Self-reported/measured height and weight; BMI classification	A large proportion had non-ideal nutritional status; 52 teachers were overweight or obese.
[51] Brazil, Pelotas	Census-based cross-sectional; 188 PE teachers	Health behaviours/knowledge; CARD risk context	Standardized questionnaire; BMI and activity questions	The study mainly assessed knowledge rather than disease; 39.1% were classified as overweight/obese and 43.6% were insufficiently active in leisure time.
[52] Costa Rica	Cross-sectional clinical dermatological study; 47 PE educators	DERM	Questionnaire; physician dermatological examination; dermoscopy	Outdoor sun exposure was substantial; dermatoheliosis, freckles, nevi and actinic keratoses/premalignant lesions were reported.
[53] Mexico, Colima	Cross-sectional; 131 PE teachers	DERM	Questionnaire on solar exposure and skin outcomes	In total, 28.2% reported sunburns; symptoms mainly involved burning/redness and affected the face and arms; dermatological monitoring was rare.
[54] Mexico	Descriptive cross-sectional; 30 PE teachers	VOC/laryngeal	Flexible nasopharyngolaryngoscopy; acoustic voice analysis	Laryngeal hyperemia was found in 96.66%; laryngeal hypokinesia in 40%; findings were interpreted as related to vocal overuse/environmental exposure.
[55] Brazil, São Paulo	Cross-sectional qualitative/questionnaire study; 10 PE teachers	VOC	Voice Symptom Scale; open questions	Seven of ten teachers exceeded the vocal-risk/dysphonia cutoff; high-intensity and constant voice use was common, with little vocal-care training.
[56] Colombia, Bogotá	Descriptive study; 10 PE teachers	MSK, WORK	School visits, survey and clinical histories	About 90% had occupational physician-diagnosed work-related osteomuscular disorder, mainly lumbar, with disability/absenteeism consequences.
[57] Argentina, Buenos Aires	Exploratory questionnaire study; 200 PE teachers	INJ/MSK, psychological pathology	Google Forms mixed questionnaire	Self-reported physical pathologies/injuries were common, but numerical reporting was inconsistent; results should be coded cautiously.
[58] Argentina, Salta	Exploratory descriptive mixed study; 185 PE teachers	MSK, VOC/RESP, AUD, skin/eye, vascular/chronic disease	Printed questionnaire; self-reported diseases over 2014–2016	A total of 84% reported at least one disease/problem; muscle, bone or joint pain, vocal/respiratory complaints, eye/skin problems, varicose veins and chronic diseases were reported.

## Data Availability

No new data were created or analyzed in this study. Data sharing is not applicable to this article.

## Supplementary Materials

The following supporting information can be downloaded at https://www.mdpi.com/article/10.3390/jcm15207838/s1. Table S1. Eighteen full-text reports were excluded at the eligibility stage. The review identified two principal exclusion categories, defined below to make the screening decisions transparent and reproducible. Table S2: Preferred Reporting Items for Systematic reviews and Meta-Analyses extension for Scoping Reviews (PRISMA-ScR) Checklist.
