# Supplementary material for: Occupational Health Complaints Among Physical Education Teachers: A Scoping Review of Musculoskeletal, Vocal, Hearing, Respiratory, Injury-Related, Cardiometabolic, and Work-Limiting Outcomes
Source: J Clin Med. 2026 Oct 11;15(20):7838. doi: 10.3390/jcm15207838 (PMC13705412; doi:10.3390/jcm15207838)
Supplement: Supplementary file 1 [file jcm-15-07838-s001.zip › jcm-4622984-supplementary table S1.pdf]

Supplementary table S1 1 Eighteen full-text reports were excluded at the eligibility stage. The review identified two principal exclusion categories, defined below to make the screening decisions transparent and reproducible.

Exclusion category	Operational definition	Typical reason for ineligibility
PE-teacher/eligible instructional-professional data not separable	The report included physical education teachers or related instructional professionals only within a mixed sample, and results for the eligible subgroup could not be extracted independently.	Health outcomes were reported only for the combined sample, preventing attribution of the findings to the eligible population.
No eligible occupational physical-health outcome	The report did not provide extractable original data on an occupational health complaint, disorder, disease, injury, or physical health risk factor within the Population–Concept–Context eligibility framework.	The report focused on pedagogy, student outcomes, professional identity, physical-activity habits, psychological constructs without a physical-health outcome, or otherwise lacked a review-eligible health endpoint.
